# Spontaneous In Situ Formation of Liposomes from Inert Porous Microparticles for Oral Drug Delivery

**DOI:** 10.3390/pharmaceutics12080777

**Published:** 2020-08-15

**Authors:** Maryam Farzan, Gabriela Québatte, Katrin Strittmatter, Florentine Marianne Hilty, Joachim Schoelkopf, Jörg Huwyler, Maxim Puchkov

**Affiliations:** 1 Division of Pharmaceutical Technology, Department of Pharmaceutical Sciences, University of Basel, Klingelbergstrasse 50, CH-4055 Basel, Switzerland; maryam.farzan@unibas.ch (M.F.); gabriela.quebatte@unibas.ch (G.Q.); katrin.strittmatter@gmx.de (K.S.); joerg.huwyler@unibas.ch (J.H.); 2 Fundamental Research, Omya International AG, Baslerstrasse 42, CH-4665 Oftringen, Switzerland; florentine.hiltyvancura@omya.com (F.M.H.); joachim.schoelkopf@omya.com (J.S.)

**Keywords:** oral drug delivery, dissolution enhancement, phospholipids, liposomes, solid dosage forms, porous microparticles

## Abstract

Despite the wide-spread use of liposomal drug delivery systems, application of these systems for oral purposes is limited due to their large-scale formulation and storage issues. Proliposomes are one of the formulation approaches for achieving solid powders that readily form liposomes upon hydration. In this work, we investigated a dry powder formulation of a model low-soluble drug with phospholipids loaded in porous functionalized calcium carbonate microparticles. We characterized the liposome formation under conditions that mimic the different gastrointestinal stages and studied the factors that influence the dissolution rate of the model drug. The liposomes that formed upon direct contact with the simulated gastric environment had a capacity to directly encapsulate 25% of the drug in situ. The emerged liposomes allowed complete dissolution of the drug within 15 min. We identified a negative correlation between the phospholipid content and the rate of water uptake. This correlation corroborated the results obtained for the rate of dissolution and liposome encapsulation efficiency. This approach allows for the development of solid proliposomal dosage formulations, which can be scaled up with regular processes.

## 1. Introduction

Liposomes are one of the most studied drug delivery systems, owing to their ability to encapsulate various active substances of hydrophobic and hydrophilic nature, their biocompatibility, and design flexibility. Although liposomes were originally developed for parenteral drug delivery, their use for oral drug delivery has been a topic of research [1,2,3,4]. Liposomes are used for oral bioavailability enhancement of low-soluble or low-permeable active ingredients [4,5,6,7], as well as compounds with low stability in the gastrointestinal (GI) tract [2,8,9,10,11].

Compared to the widely used conventional solid dosage forms (i.e., tablets), production of liposomes is demanding due to production challenges, as well as physical and chemical stability issues. The production methods are optimized for small-scale production and are unlikely to qualify for oral dosage form production [12,13]. Moreover, liposomal dispersions that are stored in liquid form have a limited stability due to the risk of sedimentation, aggregation, and phospholipid hydrolysis [14]. It would also be beneficial to produce solid powders that can be processed using conventional tableting equipment.

Freeze-drying [15,16], spray-drying [17], spray freeze-drying [18], and supercritical fluid technology [19,20] are therefore used for producing solid liposomal formulations [21,22]. One of the methods for solidification of the oral liposomes is adsorption on solid inert carriers [23]. In 1986, Payne et al. [24] introduced dry and free-flowing liposomal formulations that could produce multi-lamellar liposomes upon hydration. These systems were given the name “proliposomal formulations” and have been a subject of various studies [5,25,26,27,28,29].

As adsorption carriers for dry liposomal formulations, water-soluble carriers such as sorbitol, mannitol, or cellulose derivatives have generally been used to enhance hydration of the phospholipids [27,30,31,32,33,34]. In this study, we propose the use of a pharmacologically inert porous carrier for a single-step production of solid proliposomal formulations. Porous materials offer various benefits for drug delivery, including their large surface area and large pore volumes that allow adsorption of materials and protection of the loaded material from harsh external conditions [35]. Additionally, porous media can facilitate the hydration of liposome-forming phospholipids by rapid water uptake due to the capillary action of the porous microstructure.

In the past years, we have focused on porous functionalized calcium carbonate (FCC) microparticles as a new carrier for drug delivery. FCC is a calcium carbonate that has undergone partial recrystallization to form a variable layer of lamellae of calcium phosphate (hydroxyapatite) on the surface of the particles. The particles have an average size of 10–20 µm and exhibit a specific surface area of 30–70 m^2^/g and roughly 60% intra-particulate porosity [36]. The intra-particulate pore sizes range from 500 nm to less than 100 nm and the pores can be loaded with different materials [37]. We have harnessed the capabilities of the internal porosity of FCC particles to hold small molecular compounds [38,39] and peptides [35]. Drug loading into FCC particles allows for convenient handling and processing of compounds that would otherwise be challenging to formulate as conventional oral dosage forms. We have also studied the behavior of FCC in direct compaction and granulation processes that have resulted in FCC tablets with high tensile strength and fast disintegration [40]. Such unique mechanical characteristics of this novel porous material have allowed the production of orally disintegrating and gastro-retentive tablets from FCC [41,42,43].

The goal of the present study was to produce dry powder proliposomal formulations using the inert porous material FCC as a carrier. The feasibility of this approach was demonstrated on the basis of spontaneous formation of liposomes during hydration, drug encapsulation efficiency of the emerged liposomes, and their potential to improve the dissolution performance of nifedipine as a model low-soluble drug. Ultimately, the factors influencing the formation of liposomes and liposomal drug encapsulation were studied in different simulated gastrointestinal fluids.

## 2. Materials and Methods

FCC was kindly provided by Omya International AG (Oftringen, Switzerland), and 1,2-dimyristoyl-sn-glycero-3-phosphocholine (DMPC) was a contribution of Lipoid GmbH (Ludwigshafen, Germany). Nifedipine and methanol were supplied by Sigma-Aldrich (subsidiary of Merck KGaA, Darmstadt, Germany). Acetonitrile and fuming HCl were bought from Carl Roth GmbH (Karlsruhe, Germany). Fasted state-simulated intestinal fluid powder and buffer concentrate were purchased from Biorelevant.com Ltd. (London, United Kingdom). The solvents were of high-performance liquid chromatography (HPLC) grade, and all other materials were of analytical grade.

### 2.1. Loading of Nifedipine and DMPC in FCC

Nifedipine–DMPC–FCC formulations with various ratios of nifedipine and DMPC, as well as the corresponding control formulations, were produced by a one-step solvent evaporation method as follows: Required amounts of DMPC and nifedipine (Table 1) were dissolved in 20 mL methanol and in the final step, the sieved (355 µm) FCC powder was dispersed in this solution. The solvent was removed by evaporation in a rotary evaporator (Büchi, Switzerland). The initial pressure was set to 300 mbar and reduced by 100 mbar every 30 min. We kept the final pressure (20 mbar) for 60 min to ensure that most of the solvent was removed. The resulting drug-loaded FCC powders were then sieved through a 355 µm sieve.

Physical mixture was produced for reference purposes by manually blending the sieved (355 µm) amounts of individual components and mixing for 5 min.

### 2.2. Quantification of the Drug Content in Formulations

We quantified the nifedipine content in the formulations using a reverse-phase high-performance liquid chromatography (RP-HPLC) method. A C18 column, 90 Å, 5 µm, 3.9 × 150 mm (Resolve C18, Waters, Milford, MA, USA) was used as the stationary phase with a C18 guard column (Security Guard, Phenomenex, Torrance, CA, USA). A mobile phase composition of water/methanol/acetonitrile (50:25:25) was isocratically pumped at a flow rate of 1 mL/min. A high sensitivity flow cell for SPD-M30A diode array detector (Shimadzu, Kyoto, Japan) was used at 236 nm. The injection volume was 5 µL. Test solutions were prepared by dispersing 10 mg of the sample in 5 mL 0.1 M HCl to dissolve the calcium carbonate and calcium phosphate components of FCC. This was followed by sonicating the dispersions for 5 min to ensure complete dissolution of the carrier. A volume of 5 mL methanol was added in a subsequent step and the samples were sonicated again for 5 min. The solutions were diluted once more (1 mL to 10 mL) with the mobile phase and filtered through 0.45 µm syringe filters. All sample preparations were made in triplicate. The chromatographic measurements for each sample were carried out in triplicate. The absorbance was measured at a wavelength of 236 nm.

### 2.3. Screening for an Absence of External Crystallization

We investigated crystallization of nifedipine outside of the FCC particles by visual analysis of scanning electron microscopy (SEM) images (FEI Nova Nano SEM230, FEI, Hillsboro, OR, USA). Small amounts of the powders were placed on carbon adhesives and dedusted. The samples were sputtered with a 20 nm gold layer before imaging.

We used a focused ion beam scanning electron microscopy (FIB-SEM) method to visualize the internal structure of loaded FCC. To investigate the effect of phospholipid on nifedipine loading in FCC, we selected the formulations D5N20 and D20N20 as representative samples for lowest and highest phospholipid content, respectively, for FIB-SEM. Nif-FCC control formulation (without phospholipid) and pure FCC were used as reference material. To eliminate inter-particulate pores and visualize only the internal pore loading, we consolidated the powders with a compressive pressure of 50 MPa. Previous studies have shown that this pressure effectively eliminates inter-particulate pores without affecting the intra-particle structure [37,40]. The samples were sputtered with a 20 nm gold layer before visualization. Tilt angle of 52 degrees was used for ion beam processing and further imaging (FEI Helios Nano Lab 650, FEI, Hillsboro, OR, USA). To produce trenches with a size of 20 × 10 × 8 µm, we milled down the selected regions of the samples using a 21 nA gallium ion beam. Prior to the ion-milling step, a platinum protective layer with a thickness of 0.3 µm was deposited on the location of the trench to protect the surface of the sample from deformations during the ion beam application. After milling, the surface of the cross-section was polished with a 0.79 nA ion beam and sputtered with a 3 nm platinum layer, prior to SEM imaging, to enhance conductivity of the surface and reduce imaging artifacts.

### 2.4. Liposomal Size Measurement

Simulated gastric fluid without enzyme (SGF), pH 1.2, and phosphate buffer, pH 6.8, were produced as per United States Pharmacopeia and the National Formulary (USP-NF) [44]. The hydrodynamic size of the liposomes in SGF and phosphate buffer was measured by dynamic light scattering (DLS) using a Malvern Zetasizer Nano (Malvern Instruments, United Kingdom). For this measurement, aliquots of the powder formulations were added to the medium to result in a 5 mg/L concentration of nifedipine in the solution. The samples were stirred at 37 °C for 10 min to allow release of liposomes. The dispersions were then centrifuged at a 1000 relative centrifugal field (RCF) for 10 min at room temperature to separate FCC particles and undissolved drug from the liposomal dispersion. The DLS measurements were made on the supernatants for 10 measurement cycles.

### 2.5. Quantification of Nifedipine eEncapsulation in Liposomes

To quantify the nifedipine encapsulation efficiency of the liposomes, we dispersed the samples in the SGF and phosphate buffer and prepared them as described previously in Section 2.4. The total dissolved nifedipine in the supernatants was measured with HPLC. To separate the free drug from the liposomal-bound drug, we centrifuged the samples in 30 kDa cutoff Amicon Ultra Centrifugal Filter Units (Merck Millipore, Merck KGaA, Darmstadt, Germany) for 10 min at 4000 RCF at room temperature. The amount of the free drug and the encapsulated drug were measured by HPLC from the filtrate and the retentate, respectively, and the encapsulation efficiency of the liposomes in every formulation was calculated.

### 2.6. Liposome Imaging by Cryo-Electron Microscopy (Cryo-EM) and Fluorescent Microscopy

For cryo-EM imaging, the retentates from the centrifugal filtration were diluted with 500 µL of MilliQ water, loaded on holey carbon-coated grids (Lacey, Tedpella, Redding, CA, USA) and vitrified in liquid ethane at −180 °C using a vitrobot (FEI Company, Eindhoven, Netherlands). The frozen grids were transferred to a Talos Electron microscope (FEI, Hillsboro, OR, USA) and imaged at cryogenic temperatures using a Gatan 626 cryo-holder (Gatan, Pleasanton, CA, USA). The electron micrographs were recorded at an accelerating voltage of 200 V using a low-dose system (30 e^−^/Å^2^) and keeping the sample at −175 °C. Defocus values were −2 to −3 µm. Micrographs were recorded on a 4K × 4K Ceta CMOS camera.

For fluorescent imaging, formulations loaded with the 1,1′-dioctadecyl-3,3,3′,3′-tetramethylindocarbocyanine perchlorate (DiI) as a model hydrophobic substance were used. The samples were dispersed in a high concentration, suitable for imaging, as follows. A 20 mg aliquot of the sample was added to 50 mL of the solvent and the dispersion was shaken in a water bath at 37 °C. A droplet of the sample was visualized on a microscope slide with a cover glass using an Olympus CKX41 microscope (Olympus Ltd., Tokyo, Japan) at an excitation wavelength of 549 nm and an emission wavelength of 565 nm.

### 2.7. In Vitro Dissolution

Fasted state-simulated intestinal fluid (FaSSIF) was prepared according to the recommended recipe from the supplying company [45].

To determine the equilibrium solubility of the drug in SGF, we used phosphate buffer, pH 6.8, and FaSSIF, a saturation shake-flask method as follows: An excess amount (100 mg) of nifedipine was added to 5 mL of each buffer. The samples were protected from light and stirred in a water bath at 37 °C for 96 h. Samples were then centrifuged at 2000 RCF for 30 min and analyzed with HPLC. Each measurement was performed in triplicate.

For in vitro dissolution testing, aliquots of formulations were filled in hydroxypropyl methylcellulose capsules. The dosage of all formulations were selected to achieve a 5 mg/L concentration of nifedipine in the dissolution vessel. The dissolution experiments were carried out in a USP II apparatus (Sotax, Aesch, Switzerland) in 900 mL of medium [44] with stainless steel sinkers to prevent capsule floatation. The temperature was set to 37.1 ± 0.5 °C and the paddle speed was 100 rpm. Nifedipine concentration was measured by UV–VIS (Ultraspec 3100 pro, Amersham Biosciences, United Kingdom) at a 236 nm wavelength with 1-min software-controlled intervals (DissoControl v1.0, University of Basel, Basel, Switzerland). In parallel, to reduce errors introduced by possible phospholipid structures (i.e., lipid vesicles), for quantification of the dissolved nifedipine, we made manual samplings. Manual sampling was done after 5, 10, 15, 30, 60, 90, 120, and 150 min. To eliminate any undissolved nifedipine crystals in the dissolution aliquotes, we centrifuged an amount of 2 mL of the samples at 37 °C and 2000 RCF for 15 min. A volume of 500 µL of the supernatant was diluted with an equal volume of methanol to dissolve phospholipid vesicles. Removed volumes were replaced with fresh dissolution medium.

Assuming sink condition in the dissolution vessels, we fitted the dissolution profiles to Equation (1):

(1) $$C_{t}=C_{d}-ae^{-kt}$$

where *C_t_* (*mg/L*) is the concentration of nifedipine that is dissolved in the medium at time *t* (*s*),$\;C_{d}\;$ (*mg/L*) is the concentration of the nifedipine after the dissolution reached a plateau, a is the scaling constant, and *k* is the dissolution rate constant. In order to obtain the dissolution rate constant value for all the tested formulations, we fitted the experimental dissolution curves to Equation (1). The fittings were done in Wolfram Mathematica software, version 12 (Wolfram, Oxfrodshire, UK, 2019). To measure the goodness of fit, we used the standard errors for parameter estimates from the default Non-linear Model Fit function of the Mathematica software [46]. Two-way ANOVA and Bonferroni post-hoc test were performed (OriginPro 2018, OriginLab, Northampton, MA, USA) on the obtained dissolution rate constants with significance levels of 0.05 and 0.005, respectively.

The dissolution efficiency (*DE*) of the formulations in the first 120 min of the in vitro dissolution was calculated according to Equation (2) [47]:

(2) $$DE=\frac{\int_{0}^{120}D_{120\cdot }dt}{D_{max\cdot }120}\times 100=\frac{AUC_{0–120}}{D_{max}}\times 100$$

where $D_{120}$ is the amount of drug dissolved at 120 min (%), $D_{max}$ is the maximum drug dissolved at the end of the dissolution (%), and $AUC_{0–120}$ is the area under the dissolution curve between the times of 0 and 120 min.

### 2.8. Differential Scanning Calorimetry

A differential scanning calorimeter (DSC3, Mettler Toledo, Columbus, OH, USA) was used for thermal analysis of the samples. The scans were carried out in the interval between 30 and 200 °C with a rate of 10 °C/min, followed by an isothermal step at 200 °C for 5 min. The amount of crystalline drug can be calculated on the basis of the melting enthalpy of the drug [48]. Here, a simplified method was used [49,50] to calculate the amount of crystalline drug in FCC-Nif and D5N20 formulations:

(3) $$X_{c}\left(\%\right)=\frac{∆H_{load}}{∆H_{bulk}}\times 100$$

where $X_{c}$ is the amount of crystalline drug, $∆H_{load}$ is the melting enthalpy of the drug-loaded formulation, and $∆H_{bulk}$ is the melting enthalpy of the drug in bulk nifedipine.

### 2.9. X-ray Powder Diffraction

The solid state of the drug in the samples was investigated by X-ray powder diffraction (XRPD) with a Rigaku SmartLab diffractometer equipped with a 9 kW rotating anode and a HyPix-3000 detector (Rigaku Corporation, Tokyo, Japan). Bragg–Brentano optics were set as follows: 5.0 degree (deg) incident parallel Soller slit, 1/8 mm incident slit, 10 mm length limiting slit on the incident arm of the goniometer and 4 mm receiving slit #1, open parallel slit analyzer, 5.0 degree receiving parallel Soller slit, and 13 mm receiving slit #2 on the receiving arm of the goniometer.

The samples were exposed to X-rays of Cu wavelength (1.541 Å) and measured in 1D detection mode in a θ/2θ range of 2–60 degree with a step size of 0.01 deg and a 1 deg/min scanning speed.

### 2.10. Surface Area Measurements

The specific surface area of the samples was measured using nitrogen sorption and a five-point Brunauer–Emmet–Teller (BET) method using a Nova 2000e surface area analyzer (Quantachrome Instruments, Boynton Beach, FL, USA). All measurements were carried out in triplicate. Prior to surface area measurements, the samples were evacuated and degassed for a minimum of 3 h at room temperature.

### 2.11. Water Sorption Measurements

The rate of the water uptake of the powder samples was measured using a Krüss K100 Force Tensiometer (KRÜSS GmbH, Hamburg, Germany). The powders were loosely packed in capillaries with an inner diameter of 1 mm. As soon as the samples came into contact with water, we recorded the increase in the mass. The Lucas–Washburn equation [51,52] was used to calculate the rate of water sorption:

(4) $$\frac{l^{2}}{t}=\frac{\left(C\cdot \bar{r}\right)\sigma \;cos\;\theta }{4\eta }$$

where l (*m*) is the position of the wetting front, t (*s*) is the liquid sorption time, C is the quantity describing the orientation of the microcapillaries (*dimensionless*), $\bar{r}$ (*m*) is the average radius of the capillaries (assuming the powder porosity as a bundle of capillaries), σ (*N/m*) is the surface tension of the liquid, θ is the wetting angle (*deg*), and η (*Pa × s*) is the dynamic viscosity of the liquid [53]. The wetting constant ($C\times \bar{r})$ of each formulation was calculated by the LabDesk software (version 3.0, KRÜSS GmbH, Hamburg, Germany).

## 3. Results

### 3.1. Characteristics of the Liposomal System after Hydration of the Powder Formulations

Double chain phospholipids with a phosphatidylcholine head group have a packing parameter (ratio between the cross-sectional area of the hydrophobic region and the hydrophilic head-group) close to 1. Therefore, these lipids are likely to form bilayer structures as compared to micellar assemblies [54,55,56].

**Figure 1:**
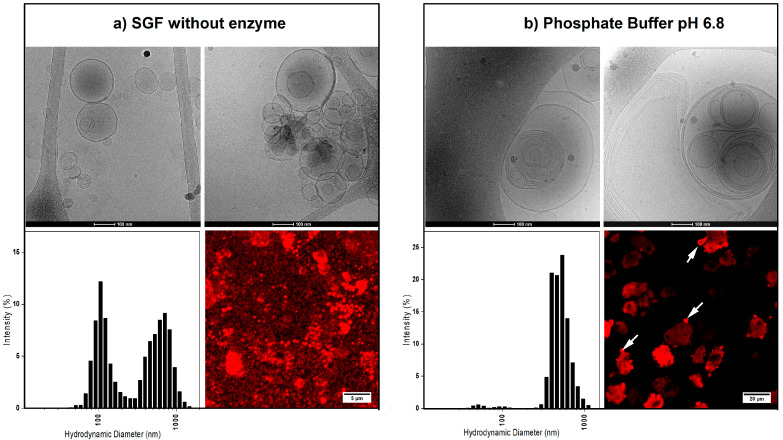
The morphology (i.e., uni-lamellar versus multi-lamellar) and size distribution of the liposomes after dispersing the powders in simulated gastric fluid without enzyme (SGF) (**a**) and phosphate buffer (**b**). For each sample, the top panels show the cryo-EM images of the resulting liposomes. The bottom left panels show the average hydrodynamic diameter of the liposomes in each medium, and the bottom right panels are the fluorescent microscopy images, showing the liposomes encapsulating a hydrophobic dye. In phosphate buffer, the liposomes can be seen emerging from the functionalized calcium carbonate (FCC) particles (white arrows).

The DLS measurement, cryo-EM imaging, and fluorescent microscopy showed the size, morphology, and the formation dynamics of the liposomes after the hydration of the formulations in the acidic condition of SGF and the neutral condition of phosphate buffer (Figure 1, Figure S1).

The hydrodynamic diameter of the liposomes in SGF and phosphate buffer showed a population at 669 ± 169 nm and 490 ± 112 nm average size, respectively. However, in SGF, the liposomes additionally had a second population with an average size of 121 ± 36 nm (Figure 1, lower left panels). No difference was observed between the liposome sizes of different formulations (data not shown). Cumulant analysis of the samples obtained a z-average of 261 ± 83.07 (polydispersity index 0.56 ± 0.13) and 420 ± 86.86 nm (polydispersity index 0.60 ± 010) for the liposomes in SGF and phosphate buffer, respectively.

The cryo-EM imaging of the liposomes corroborated the DLS results by showing many uni-lamellar and a small number of multi-lamellar liposomes after dissolving in SGF. On the other hand, the liposomes in the phosphate buffer had larger sizes and mostly multi-lamellar and multi-vesicular morphologies.

Fluorescent imaging showed incorporation of the hydrophobic fluorescent dye (DiI) in the lipid bilayer of the liposomes. For samples prepared in SGF, almost no residuals of FCC particles could be observed. The supplementary optical microscopy video (Video S1) shows the dissolution process of FCC particles in SGF and formation of the liposomes. Formation of CO_2_ bubbles resulting from the dissolution of calcium carbonate is clearly seen on the video. On the other hand, dispersion of the samples in phosphate buffer showed that FCC particles remain intact, while liposomes of various shapes and sizes slowly emerge out of the porous structure of the particles.

**Figure 2:**
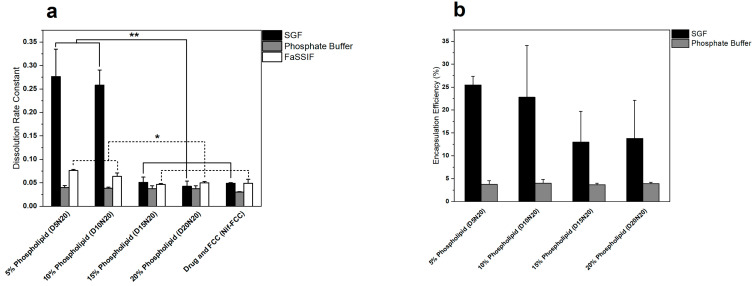
Comparison of the dissolution rate and liposomal encapsulation efficiency of nifedipine for different formulations. (**a**) The dissolution rate of formulations obtained from fitting the dissolution data to Equation (5), and (**b**) encapsulation efficiency of the liposomes after the hydration of powders in different media. Increasing the phospholipid content inversely affected both the dissolution rate and encapsulation efficiency. Single asterisk indicates a *p*-value < 0.05 and the double asterisk indicates a *p*-value < 0.005.

The encapsulation efficiency of nifedipine (Figure 2b) was significantly greater in the liposomes created in SGF, as compared to phosphate buffer. The encapsulation efficiency was generally higher in the formulations with lower phospholipid contents (5% and 10%) at a maximum average of 25% for D5N20. However, the encapsulation efficiency was not significantly different among the formulations in the same dissolution medium (*p*-value > 0.05).

### 3.2. In Vitro Dissolution

Nifedipine equilibrium solubility in SGF, phosphate buffer, and FaSSIF were 17.94 ± 2.21 mg/L, 16.06 ± 1.71 mg/L, and 48.86 ± 25.26 mg/L, respectively. Therefore, the 5 mg/L drug concentration for in vitro dissolution tests was significantly below the saturation solubility of nifedipine in all three media, thus implying sink condition.

**Figure 3:**
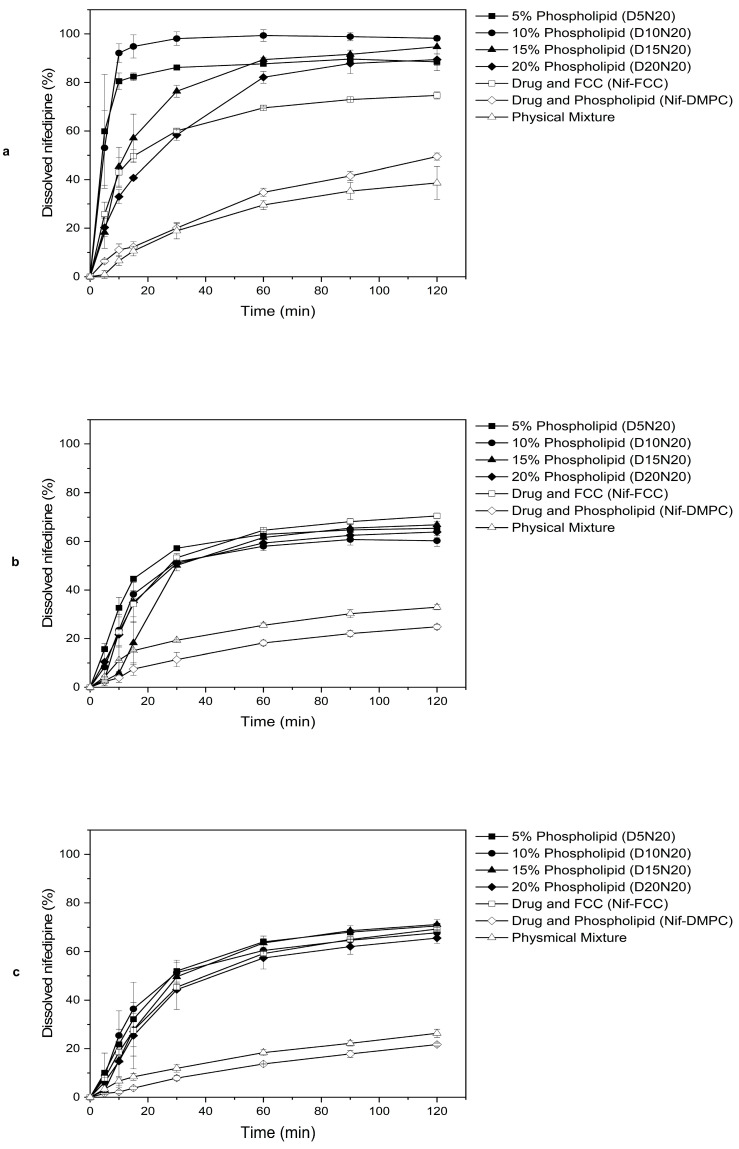
Selected FCC formulations (see Table 1) tested for dissolution in three different dissolution media: (**a**) SGF without enzyme, (**b**) fasted state-simulated intestinal fluid (FaSSIF), and (**c**) phosphate buffer, pH 6.8. Solid symbols represent formulations containing an increasing amount of phospholipid. Open symbols represent control formulations. Values are mean ± SD of *n* = 2 or 3 tests.

For all media, the dissolution rate of nifedipine was faster for the formulations with FCC than for the reference formulation without FCC (Nif-DMPC), as well as for the physical mixture (Figure 3).

The dissolution results in SGF showed that the rate of dissolution of nifedipine was inversely proportional to the phospholipid content. The changing ratio of drug to phospholipid did not have any influence on the rate of dissolution (Table S1). Therefore, all further analyses were carried out on the formulations with the highest drug load (20%) and with varying phospholipid content (i.e., D5N20, D10N20, D15N20, and D20N20).

The 5% and 10% phospholipid formulations showed significantly faster drug release in SGF, compared to the formulations with 15% and 20% phospholipid content, and Nif-FCC (Figure 2a and Figure 3a). All formulations with DMPC and FCC reached at least 85% drug dissolution.

None of the formulations reached 85% dissolution (Figure 3b) in FaSSIF. However, the rate of dissolution was significantly faster for the formulations with 5% and 10% phospholipid content (Figure 2a).

Similar results were obtained in the phosphate buffer, where none of the formulations reached 85% dissolution (Figure 3c). The rate of dissolution in the phosphate buffer was not significantly different within the formulations with FCC (*p*-value > 0.05).

The dissolution efficiency (DE) of formulations, as a measure of the dissolution behavior of the formulations, are shown in Table 2.

The results show a reduction in the DE of Nif-DMPC in all media. In SGF, a trend of reduction in DE was seen with increasing phospholipid contents.

### 3.3. Characterization of the Dry Powder Formulations

**Figure 4:**
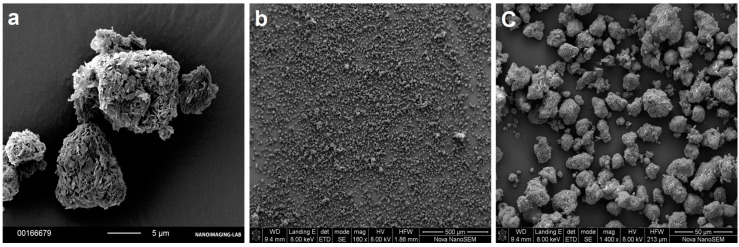
SEM images used for visual analysis of drug-loaded FCC. (**a**) The surface lamella of the unloaded FCC particles can be seen. (**b**) Representative overview of the powder formulation with highest loaded material (D20N20), showing the absence of agglomerates and external crystals. (**c**) Formulation D20N20 showing preserved lamellar structure of FCC after loading.

As an assessment of the loading efficiency, we visually investigated the appearance of the particles using SEM. The obtained images showed individual particles with neither agglomerates nor external drug crystallization (Figure 4b). There was no observable blocking or covering of the particle surfaces (Figure 4c). The images of the loaded samples were similar to the reference FCC material (Figure 4a).

**Figure 5:**
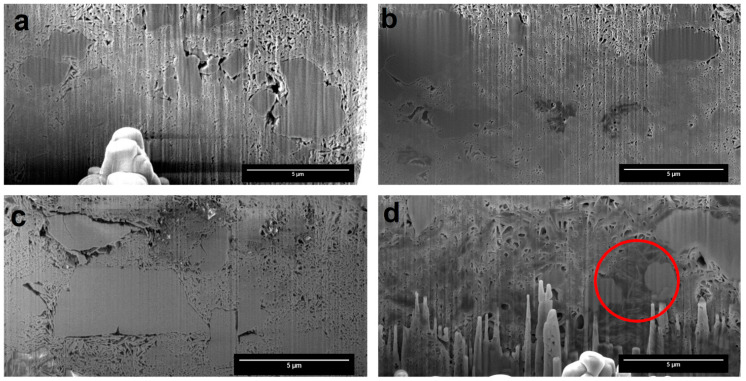
The focused ion beam scanning electron microscopy (FIB-SEM) images showed cross-sections of the formulations after consolidating the powder. (**a**) Pure FCC, (**b**) Nif-FCC without phospholipid, (**c**) D5N20, and (**d**) D20N20. The red circle shows an example of a region with blocked pores.

FIB-SEM images from the cross-section of the particles (Figure 5a) showed intra-particle larger pores evenly distributed throughout the entire FCC compact [40], while the drug-loaded formulations had smaller pores. The formulations Nif-FCC (Figure 5b) and D5N20 (Figure 5c) were not visually different, neither in pore sizes nor in pore shapes. On the contrary, the filled porous structure was seen for the formulation D20N20 (Figure 5d).

A loading efficiency of above 90% for the drug in all of the powder formulations was measured.

**Figure 6:**
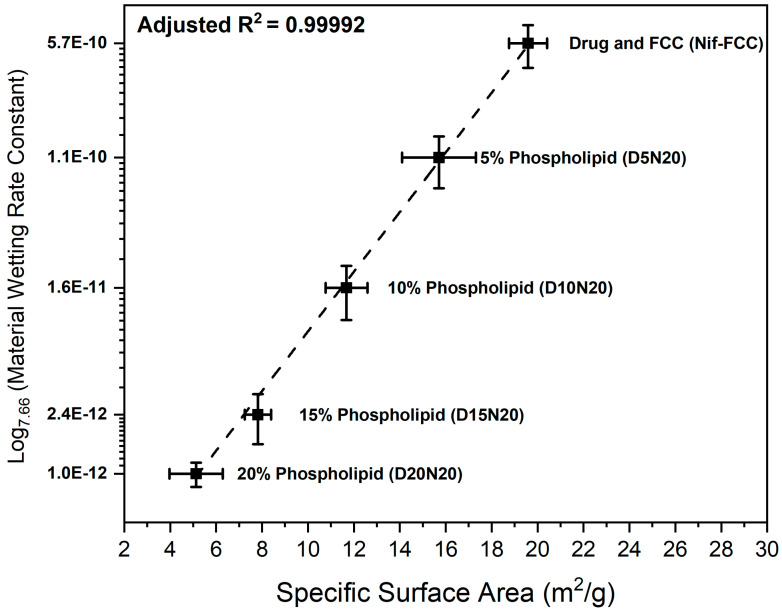
The semi-logarithmic plot (base 7.66) of the correlation between the wetting constants versus specific surface area of the powder samples.

On the basis of the results of BET surface area measurements, the specific surface area of the formulations decreased due to an increase in the phospholipid loading (Figure 6).

The water sorption measurements showed an inverse correlation between the rate of water uptake and phospholipid content (Figure 6).

Figure 6 shows the correlation of the power law (represented as a semi-logarithmic plot) of the material wetting constant (obtained from Equation (4)) with the specific surface area of the formulations. For this correlation, the experimental data were fitted to Equation (5):

(5) $$S=aC^{n}+b$$

where *S* is the specific surface area (*m*^2^); *C* is the wetting constant obtained from Equation (4); a is the scaling parameter, intercept b→0; and exponent n=7.66 are the parameters obtained after fitting the experimental data to Equation (5). The two sets of results showed a strong positive power law correlation (adjusted *R*^2^ = 0.99992).

**Figure 7:**
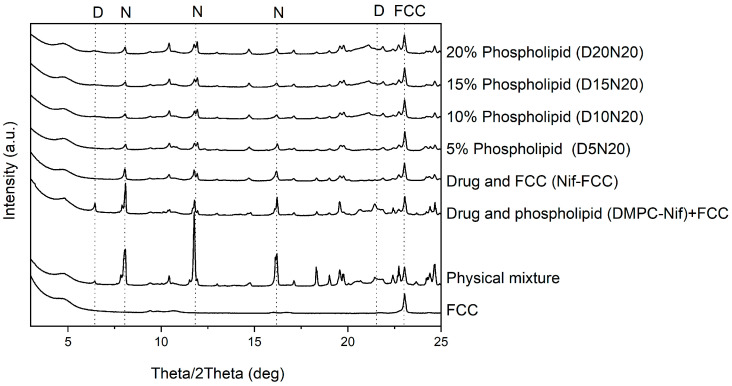
XRPD results of the formulations and physical mixture. The model drug was present in partial crystalline form in all formulations. The reference peaks of nifedipine (N) (8.11°, 11.76°, and 16.16°) and 1,2-dimyristoyl-sn-glycero-3-phosphocholine (DMPC) (D) (6.65° and 20.98°) are marked with dashed lines. A reference peak for FCC at 23.01° is also marked. The sample DMPC-Nif was physically mixed with FCC to keep the ratio of the components constant for all samples.

X-Ray powder diffraction was used to exclude an effect of complete drug amorphization. The X-ray diffraction results of samples are presented in Figure 7. The control formulations in the absence of phospholipid or FCC both showed strong characteristic peaks of nifedipine at 8.11°, 11.76°, and 16.16°. The intensity of these peaks was lower in the formulations with DMPC and FCC.

The results of thermal analysis (Figure S2) showed the nifedipine melting peak at 172 °C. However, the samples with 5% phospholipid (D5N20) and without phospholipid (Nif-FCC) showed a reduced melting enthalpy for nifedipine. On the basis of calculations according to Equation (3), we detected crystallinity ratios of 60.76 ± 0.69% and 57 ± 9.91% for the D5N20 and Nif-FCC samples, respectively. The nifedipine melting peak was not present in the samples prepared with higher content of phospholipid.

## 4. Discussion

The results of the present study showed massive spontaneous in situ formation of liposomes from a porous inorganic drug carrier combined with phospholipids. This effect was observed in media simulating the conditions of the different regions of the GI tract. As a consequence, this effect led to dissolution rate enhancement of a model low soluble drug.

A successful drug loading in the internal pores of the carrier is essential for effective porous structure exploitation. In this study, the loading efficiency was visually controlled. Absence of external deposition of drug crystals and phospholipid material was confirmed by visual image analysis for every formulation (excluding physical mixtures), as shown in Figure 4. The results suggest that the phospholipid and nifedipine were loaded in the internal structure of the porous FCC particles. This result was corroborated by FIB cross-sectioning of the consolidated and loaded FCC, as shown in Figure 5. By visual analysis of internal porosity reduction, as clearly seen on the FIB-SEM images (Figure 5d), we could confirm the loading of drug and phospholipid into internal carrier structures. The results of BET measurements (Figure 6) showed a reduction in specific surface area, which further confirmed the hypothesis of internal (i.e., intraparticulate) deposition of the loaded material.

Both calcium carbonate and calcium phosphate, the main constituents of FCC, readily dissolve in the low pH of the gastric medium, which triggers an immediate release of liposomes (Video S1). On the other hand, in neutral pH media, the formation of liposomes from FCC particle followed a different mechanism. Since the carrier was not immediately dissolved in the neutral pH, the liquid was taken up into the particles by the carrier’s micro-capillaries. This observation suggests a capillary pump action for the porous microstructure of FCC. The capillary pump effect supposedly drives the hydrated phospholipids out of the pores and promotes self-assembly of liposomes. This process can be seen in the confocal microscopy images, revealing the liposomes emerging (budding) out of the pores of FCC (Figure 1).

In SGF (i.e., medium with a low pH), a significantly large population of the liposomes had a size of 121 ± 36 nm and were observed as uni-lamellar structures in cryo-EM images. This size range was possibly due to fast release of liposomes from FCC, aided by the eddy currents created as a result of CO_2_ formation from calcium carbonate dissolution in the acidic pH. The liposomes formed in this process were able to encapsulate up to 25% of the nifedipine, resulting in a faster dissolution of the drug compared to the drug release rate from the formulation without phospholipids. The different liposome formation mechanism in neutral pH produced large multi-lamellar liposomes (490 ± 112 nm) as seen in the cryo-EM images. These liposomes had a lower encapsulation efficiency, as compared to the uni-lamellar liposomes in SGF. This finding was surprising since the multi-lamellar liposomes were expected to have a higher encapsulation efficiency for hydrophobic drugs compared to uni-lamellar liposomes. The low encapsulation efficiency in neutral media was explained by the slow rate of formation and release of the liposomes from FCC particles, which allowed enough time for the drug to escape from the liposomes before their detachment from the carrier. This assumption was further confirmed by the results that showed a similar rate of dissolution of nifedipine from FCC phospholipid formulations and the Nif-FCC (the formulation without phospholipid) in neutral pH. In other words, in neutral pH, nifedipine dissolution takes place independently of the liposome formation, and the dissolution rate is enhanced solely by the large specific surface area of the carrier.

Due to the surface-active properties of phospholipids, faster dissolution rates are expected from formulations with higher DMPC content. Despite the latter, an increased phospholipid content resulted in slower dissolution rates in SGF (Figure 2 and Figure 3). The higher phospholipid amounts in FCC reduce the overall surface area available for drug release by blocking out the carrier’s porous meshwork, which leads to a reduction in the surface available for dissolution. This effect was confirmed by the results of BET surface area experiments, where a reduction of specific surface area with an increase in the phospholipid content was measured. For example, the D5N20 sample with 5% phospholipid content had a threefold larger specific surface area and an almost sevenfold faster dissolution as compared to D20N20.

The importance of the available medium-to-solid contact surface is emphasized since FCC itself dissolves in the low pH dissolution medium. This is forcing single particles to disintegrate, which leads to a faster release of the liposomes that can better encapsulate the drug (i.e., not letting sufficient time for the drug to escape the forming liposomes) and results in a faster rate of dissolution. Additions of more phospholipid lead to a slower wetting, confirmed by the water sorption studies, and therefore slower reaction of FCC with the medium. This makes the drug release dependent on the diffusion of the drug from the carrier’s pores. A complete dissolution of calcium carbonate in SGF results in disappearance of the carrier particles; therefore, the only microclimate pH change may be expected within the formed liposomes. In theory, a change in the dissolution kinetics of the drug is expected to be negligible due to close proximity of undissolved drug crystals to the lipophilic layer of the phospholipids.

The high surface area of FCC itself effectively exposes the deposited drug to the medium and, even without phospholipids, leads to a faster dissolution of nifedipine from the FCC formulations compared to the physical mixture (Figure 3, Nif-FCC vs. physical mixture).

The X-ray diffraction and DSC results did not exclude the fact that the nifedipine might be partially deposited in a non-crystalline form in the carrier. The DSC thermograms of FCC formulations showed a reduced melting enthalpy, which was possibly a result of partial amorphization of the drug (Figure S2). Moreover, the X-ray diffractograms clearly indicated the crystalline state of nifedipine in all formulations (Figure 7); therefore, an enhanced dissolution cannot be solely attributed to the drug amorphization by phospholipids.

The wetting constant and the specific surface area depend on each other according to Equation (5), which represents the power law function (Figure 6). The reduction of the individual area of a pore’s cross section depends on squared diameter of remaining pore opening as follows from simple geometric considerations. Thus, under an assumption of constant rate of capillary transport, there is a quadratic dependency of the porous cross-section area on the rate of material deposition in a material with idealized equisized pore geometries. However, for different pore size distributions in real materials, the power exponent is expected to be significantly different from two. Therefore, due to loading of materials into the FCC particles, the smaller pores are filled first and the total liquid throughput (i.e., water uptake) through the particle meshwork changes according to power law with exponents greater than two. The applicability of the power law correlation can be extended to the dissolution rate and liposomal encapsulation efficiency as a function of the phospholipid content in the FCC-based formulations. The latter suggests a guidance for the design or section of porous carriers for such formulations.

On the basis of the results, we could show that the combination of porous FCC microparticles and phospholipids can be used to produce solid formulations oral liposomal delivery. This method is suitable for classical scale-up. We demonstrated that the fast water uptake and dissolution of the carrier is essential for achieving the highest encapsulation efficiency and the fastest drug dissolution rate.

## 5. Conclusions

As a general principle, the in situ formation of liposomes from a combination of phospholipids and porous carriers in the simulated GI conditions is an effective approach for formulating liposomes for oral delivery. We propose that a single-step loading of phospholipids and drug substances can be used as a simple and effective method for the production of proliposomal formulations. This method is suitable for scale-up. We showed the mechanism of the spontaneous in situ formation of liposomes in different dissolution media and demonstrated drug encapsulation in the emerging liposomes. To enhance the encapsulation efficiency and reduce drug leakage from the liposomes, other types of phospholipids and their combinations with amphiphilic polymers can be used with porous microparticulate carriers, such as FCC.

## Figures and Tables

**Table 1:** Expected drug and lipid composition of the sample formulations.

Formulation Code	DMPC (*w/w*%)	Nifedipine (*w/w*%)	FCC (*w/w*%)
D5N10	5	10	85
D5N15	5	15	80
D5N20	5	20	75
D10N10	10	10	80
D10N15	10	15	75
D10N20	10	20	70
D15N10	15	10	75
D15N15	15	15	70
D15N20	15	20	65
D20N10	20	10	70
D20N15	20	15	65
D20N20	20	20	60
Nif-FCC (control)	0	20	80
DMPC-Nif (control)	50	50	0
DMPC-FCC (control)	20	0	80
Physical mixture	20	20	60

**Table 2:** Dissolution efficiency of formulations calculated based on Equation (2).

Formulation Code	SGF	Phosphate Buffer	FaSSIF
D5N20	91.53 ± 1.33	79.66 ± 4.33	86.40 ± 1.33
D10N20	89.74 ± 2.96	79.35 ± 1.31	84.88 ± 1.02
D15N20	84.23 ± 1.06	76.26 ± 2.19	78.09 ± 1.10
D20N20	77.45 ± 1.37	74.95 ± 6.76	80.34 ± 0.005
Nif-FCC	84.19 ± 1.13	73.66 ± 1.95	80.19 ± 2.01
Nif-DMPC	60.12 ± 9.79	57.23 ± 1.42	64.80 ± 3.25

## Supplementary Materials

The following are available online at https://www.mdpi.com/1999-4923/12/8/777/s1, Figure S1: Cryo-EM images of liposomes. Figure S2: Differential scanning calorimeter (DSC) spectra of the formulations and physical mixture. Table S1: The time required for 80% of the drug content to dissolve in SGF. Video S1: Formation of liposomes in SGF.
