# Supplementary material for: Spontaneous In Situ Formation of Liposomes from Inert Porous Microparticles for Oral Drug Delivery
Source: Pharmaceutics. 2020 Aug 15;12(8):777. doi: 10.3390/pharmaceutics12080777 (PMC7465306; doi:10.3390/pharmaceutics12080777)
Supplement: Supplementary file 1 [file pharmaceutics-12-00777-s001.pdf]

pharmaceutics

MDPI

# Supplementary Materials: Spontaneous In Situ Formation of Liposomes from Inert Porous Microparticles for Oral Drug Delivery

Maryam Farzan [1], Gabriela Québatte [1], Katrin Strittmatter [1], Florentine Marianne Hilty [2], Joachim Schoelkopf [2], Jörg Huwyler [1] and Maxim Puchkov [1,*]

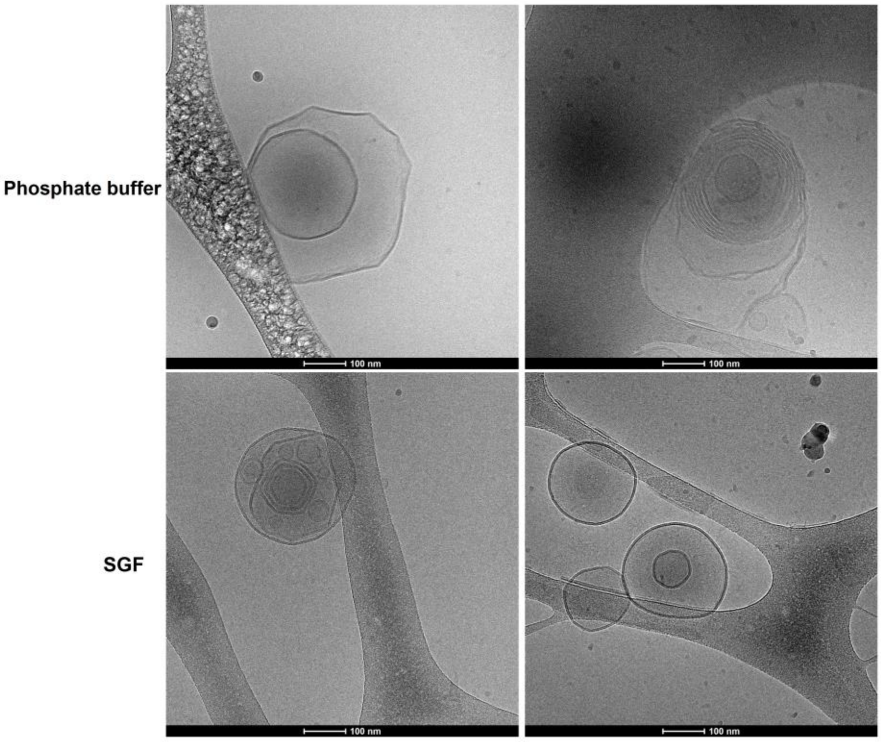


**Figure S1.** Cryo-EM images showing the liposomes created in phosphate buffer and in SGF.

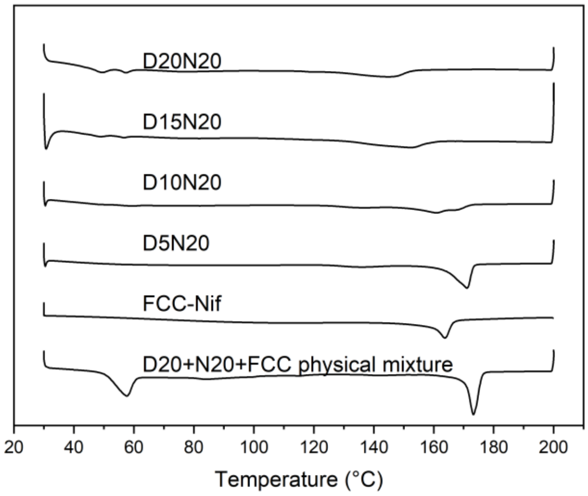


**Figure S2.** DSC spectra of the formulations and physical mixture. The nifedipine melting peak is present at 172 °C. For the formulations with greater phospholipid content (10%, 15%, and 20%) a

melting peak for nifedipine cannot be detected, which is due to the melting of the phospholipid and consequent dissolution the crystalline portion of nifedipine at a temperature below its melting point.

**Table S1.** The time required for 80% of the drug content to dissolve in SGF.

| Formulation Code | Time to 80% Dissolution (min) |
|---|---|
| D5N10 | 16.0 ± 2.8 |
| D5N15 | 15.6 ± 3.0 |
| D5N20 | 8.3 ± 3.05 |
| D10N10 | 12.8 ± 4.9 |
| D10N15 | 24.3 ± 8.5 |
| D10N20 | 8.0 ± 1.0 |
| D15N10 | 34.5 ± 11.5 |
| D15N15 | 23.6 ± 4.5 |
| D15N20 | 35.0 ± 4.5 |
| D20N10 | 68.5 ± 31.8 |
| D20N15 | 56 ± 12.2 |
| D20N20 | 48.66 ± 4.9 |

**Video S1: Formation of liposomes in SGF.**

The video has been uploaded in an external data repository. https://doi.org/10.7910/DVN/2LXYW1

## References

1. J.M. Ting, T.S. Navale, F.S. Bates, T.M. Reineke, Design of Tunable Multicomponent Polymers as Modular Vehicles To Solubilize Highly Lipophilic Drugs, Macromolecules. 47 (2014) 6554–6565. https://doi.org/10.1021/ma501839s.
2. I. Vitez, Utilization of DSC for Pharmaceutical crystal form quantitation, Journal of Thermal Analysis and Calorimetry. 78 (2005) 33–45. https://doi.org/10.1023/b:jtan.0000042151.60566.0f.
3. B. Shah, V.K. Kakumanu, A.K. Bansal, Analytical techniques for quantification of amorphous/crystalline phases in pharmaceutical solids, Journal of Pharmaceutical Sciences. 95 (2006) 1641–1665. https://doi.org/10.1002/jps.20644.
